# Late Dental Toxicities After Proton Chemoradiation for Rhabdomyosarcoma: A Pediatric Case Report

**DOI:** 10.14338/IJPT-22-00011.1

**Published:** 2022-11-16

**Authors:** Emma Foster-Thomas, Marianne Aznar, Daniel Indelicato, Shermaine Pan, Eunji Hwang, Peter Sitch, Keith Horner, Ed Smith, Simona Gaito

**Affiliations:** 1 Restorative Dentistry, University Dental Hospital of Manchester, Manchester University NHS Foundation Trust, Manchester, UK; 2 Adaptive Radiotherapy, University of Manchester Division of Clinical Cancer Science, School of Medical Sciences, Manchester, UK; 3 Department of Radiation Oncology, University of Florida Health Proton Therapy Institute, Jacksonville, FL, USA; 4 Clinical Oncology, The Christie NHS Foundation Trust, Manchester, UK; 5 Department of Radiation Oncology, Crown Princess Mary Cancer Centre, Westmead Hospital, Westmead, Australia; 6 Christie Medical Physics and Engineering, The Christie NHS Foundation Trust, Manchester, UK; 7 Dental and Maxillofacial Radiology, University Dental Hospital of Manchester, Manchester University NHS Foundation Trust, Manchester, UK; 8 The Christie Proton Clinical Outcomes Unit/The University of Manchester Division of Clinical Cancer Science, School of Medical Sciences, Manchester, UK

**Keywords:** dental toxicity, late effects, pediatric, head and neck cancer, proton beam therapy, chemotherapy

## Abstract

**Purpose:**

Radiation therapy is an independent risk factor for adverse sequelae to the oral cavity and dentition in childhood cancer survivors. However, dental toxicities after radiation therapy often are underreported and there are minimal published data on disturbances in tooth development after proton beam therapy (PBT). We present the long-term clinical and radiographic dental findings 8 years after treatment completion for a patient treated with PBT and chemotherapy for rhabdomyosarcoma.

**Materials and Methods:**

Clinical follow-up data of patients treated with PBT within the Proton Overseas Programme (POP) is stored in a National Database and curated by a dedicated outcomes unit at the Christie NHS PBT center. This case report was identified from the extraction and analysis of data for pediatric head and neck cancer patients in this database for a service evaluation project.

**Results:**

The permanent dentition in this patient aged 3.5 years at the time of treatment was severely affected with abnormal dental development first observed 3.5 years after treatment completion. PBT delivered mean doses of 30 Gy(RBE = 1.1) to the maxilla and 25.9 Gy(RBE = 1.1) to the mandible.

**Conclusion:**

Significant dental development abnormalities occurred in this pediatric patient, despite doses in areas being lower than the proposed thresholds in the literature. Improved descriptions of dental toxicities and routine contouring of the maxilla and mandible are needed to correlate dosimetric data. The dose to teeth should be kept as low as reasonably possible in younger patients until the dose thresholds for dental toxicities are known.

## Introduction

Radiation therapy is important in managing pediatric head and neck cancers [1]. As treatment protocols and outcomes improve, efforts to minimize late adverse effects must improve to optimize cancer survivorship care [2, 3]. Proton beam therapy (PBT) may reduce radiation dose to adjacent normal tissues, thus reducing treatment-related morbidity [3]. However, evidence linking specific dose thresholds to toxicities in children is lacking, particularly regarding dentofacial effects.

Radiation is known to cause damage in susceptible phases of the cell cycle of ameloblasts (enamel-forming cells) and odontoblasts (dentine-producing cells) [2]. Radiation therapy and chemotherapy during the matrix deposition and calcification phases of tooth development may result in microdontia, enamel hypoplasia, and arrested root development [2, 4].

The nature and severity of potential dental development abnormalities (DAs) are inversely related to age and tooth development stage at the time of photon radiation [5–7]. In addition, radiation doses, treatment schedules, and the concomitant use of multiagent therapy influence incidence [8–10]. Incidence of DAs is thought to increase in a dose-dependent manner, with an odds ratio of 5.6 for doses ≥ 20Gy in conventional photon radiotherapy [1]. The Pediatric Normal Tissue Effects in the Clinic review, which focuses on photon radiation, recommends that the dose to teeth should be kept “As Low As Reasonably Achievable” in younger patients, restricting doses to < 20 Gy(D_mean_) in patients < 4 years old [1].

Recognizing the paucity of literature published on dental toxicities after PBT, this case report aimed to present the long-term clinical and radiographic DAs identified in a rhabdomyosarcoma (RMS) survivor treated with PBT and chemotherapy.

## Methods and Patient Outcome Data

### Proton Overseas Programme

The Proton Overseas Programme (POP) was launched in 2008, allowing eligible patients referrals and funding via the National Health Service (NHS) for PBT overseas [3]. United States and European centers established clinical referral pathways and collaborative relationships. After treatment, patients received follow-up care at their referring UK center. Clinical follow-up data and detailed information about “baseline toxicities” have been prospectively collected on patients treated with PBT since the POP's inception and are now stored centrally in a national database managed by the Proton Clinical Outcomes Unit at The Christie NHS PBT center [11].

#### Population

From June 2008 to October 2019, the POP referred 195 pediatric head and neck cancer patients (aged 0–15 years) from multiple UK centers. Dental toxicity data (moderate to severe) were extracted from patient records via the Proton Clinical Outcomes Unit to conduct a retrospective service evaluation. The *Common Terminology Criteria for Adverse Events v4.0* defines seven toxicities related to dental structures [12]. For this service evaluation, the toxicity endpoint reviewed was “tooth development disorder” [12]. After a median follow-up of 32.8 months from the end of treatment to the last follow-up (range, 1.6–105.2 months), 4 patients were reported to have late dental toxicities. After reviewing the clinical records and available dental radiographs, DAs could only be confirmed in 1 patient. A description of alphanumeric tooth notation used throughout this report, along with definitions of DAs, are outlined in **Table 1** [13, 14].

**Table 1.** Definitions of dental development disturbances and dental terms used in this case report [13,14]

Microdontia	Abnormality of the size of the crown of a tooth. A microdont is a tooth that is smaller than normal.
Hypodontia	Absence/failure of formation of up to 6 teeth
Molar-incisor hypomineralization	A defect in the dental enamel affecting the central incisors and first molars in the permanent dentition.
Arrested root development	Premature arrest of development of the roots of a tooth.
Physiological root resorption	Resorption of the roots of the primary teeth, which normally occurs as the below permanent successor tooth erupts.
Partially erupted tooth	A tooth that has not fully emerged through the gums and is not in its final position.
Unerupted tooth	A tooth that has not yet emerged through the gums. An unerupted tooth may or may not erupt.
Alphanumeric tooth notation	The 4 quadrants of the mouth are referred to as upper right (UR), upper left (UL), lower right (LR), and lower left (LL). The permanent teeth are assigned a number from 1–8, with 1 being the central incisor and 8 the third molar. The primary teeth are numbered from A to E, with ‘A' being the central incisor and ‘E' the second molar.

### Proton beam therapy

**Figure 1.**
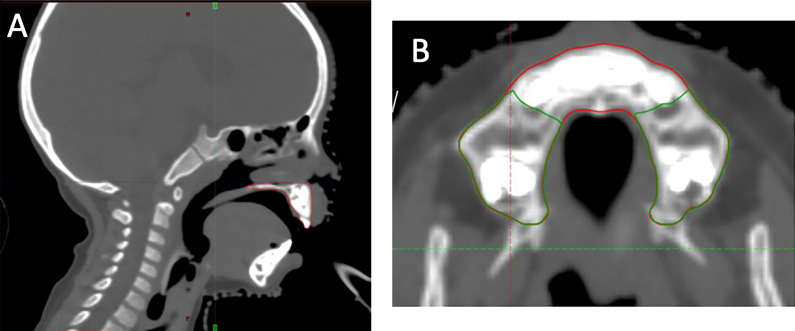
(A) The maxilla was retrospectively contoured to estimate the dose delivered. (B) The maxilla was segmented into central (red) and lateral (green) sextants.

The radiation dose to the maxilla and mandible was determined using a manual contouring approach [15]. The mandible was outlined from the alveolar crest to the inferior cortex and superiorly up to the temporomandibular joint. The mandible was divided into left and right rami (including the angle, coronoid, and condylar processes) and body (**Figure 1A**). The maxilla was outlined superiorly to the upper limit of the hard palate to exclude the maxillary sinus, inferiorly to include the tuberosities, laterally to the zygomatic arch, and posteriorly along the posterior palatine border (**Figure 1B**). Both the mandibular body and maxilla were further divided into central (defined laterally by the distal aspect of the canine teeth), left and right lateral sextants [16]. Cumulative dose-volume histograms (DVH) were produced to extract dosimetric parameters.

## Case Report

**Figure 2.**
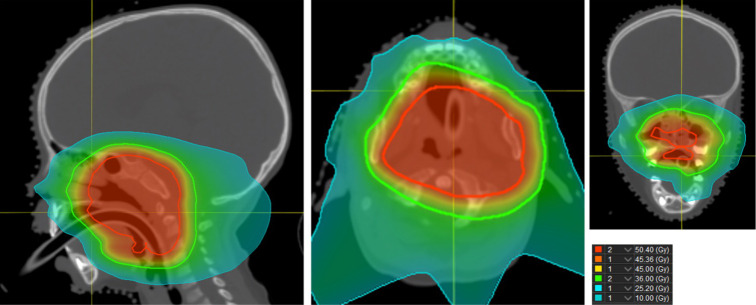
Proton beam therapy treatment plan highlighting dose distribution, as depicted by the color key, to the maxilla and mandible for treatment of an embryonal rhabdomyosarcoma of the nasopharynx. Doses given in Gy(RBE = 1.1). For example, the area depicted in red received a mean of 50.5 Gy.

A 3.5-year-old female diagnosed with embryonal RMS of the nasopharynx (T2aN0M0) was treated on the high-risk RMS 2005 protocol with 4 cycles of IVADo (I, ifosfamide; V, vincristine; A, actinomycin-D; D, doxorubicin) and 5 cycles of IVA chemotherapy [17] over 25 weeks at The Royal Manchester Children's Hospital. PBT was started on week 13 and delivered a total dose of 50.4 Gy(RBE = 1.1) in 28 fractions at the University of Florida Health Proton Therapy Institute (**Figure 2**). DAs were first recorded 3.5 years posttreatment, after a complaint of sensitivity with the hypomineralized upper right and left first permanent molars (UR6 and UL6).

To date, this patient has more than 8 years of dental follow-up, allowing the reporting of longitudinal images. It is the opinion of the authors (EFT and KH) that the developmental stage of the permanent dentition is reasonably consistent with chronological age. **Table 2** summarizes the long-term radiographic findings.

**Table 2.** A summary of the radiographic findings for this patient with corresponding figures.

Dental radiograph taken	Age of patient	Corresponding figure	Summary of radiographic findings
Orthopantomogram (OPT; extraoral bitewing setting*)	6 y 11 mo	3A	Microdontia of the UR5, UL5, LL7, LR5, and LR7.
			Hypodontia of the UR7, UL7, LL5.
			Arrested root development of all the first permanent molars with short tapering roots on the LR6.
OPT	7 y 10 mo	3B	Further root development of the unerupted mandibular permanent premolars evident—LL4, LR4, and LR5.
			Arrested root development of the maxillary incisors has resulted in short, tapered (diminished volume) hypoplastic morphology.
OPT	8 y 11 mo	3C	Normal physiological root resorption of the remaining 3 primary first molars—URD, LLD, and LRD.
			UR4 has erupted despite the extremely short root.
			No further root development is evident on the maxillary canines or the unerupted UR5.
			No sign of developing third molars.
Anterior maxillary periapical radiographs	12 y 4 mo	4	UR3 remains unerupted.
			UL3 has partially erupted, despite no further root development.
			Very short tapering roots UR2, UR1, UL1, and UL2.

**Figure 3.**
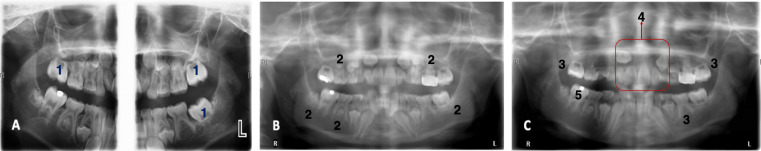
A series of dental panoramic radiographs taken following treatment completion at different age timepoints (A) 6 years 11 months, (B) 7 years 10 months, and (C) 8 years 11 months. Dental abnormalities are labelled 1 to 5 as follows: (1) Arrested root development of UR6, UL6, and LL6; (2) microdontia UR5, UL5, LL7, LR5 and LR7; (3) failure of development UR7, UL7, and LL5; (4) severe hypoplasia of roots (upper anterior teeth); and (5) mild hypoplasia of LR6 roots.

**Figure 4.**
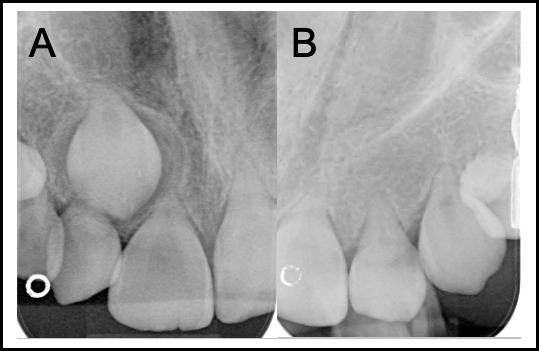
Anterior periapical radiographs taken 8.5 years after treatment showing the short tapering root form of the UR3, UR2, UR1, UL1, UL2, and UL3. (A) Periapical radiograph maxillary right anterior segment. (B) Periapical radiograph maxillary left anterior segment.

The developmental stage of the teeth in **Figure 3A** suggests an abrupt halt to root development coincident with the timing of treatment. Without pretreatment dental imaging, it is impossible to confirm whether the hypodontia is associated with the patient's treatment. However, no DAs were recorded at the pretreatment dental screening, and the absence of the LL5 tooth bud on an MRI scan taken 6-months posttreatment completion suggests congenital tooth absence. The poor prognosis of the anterior maxillary teeth is evidenced in **Figure 4**. In addition to the radiographically evidenced DAs, the UR1 and UR2 have molar–incisor hypomineralization. These teeth have a poor prognosis and tooth replacement options will need to be considered soon.

**Figure 5.**
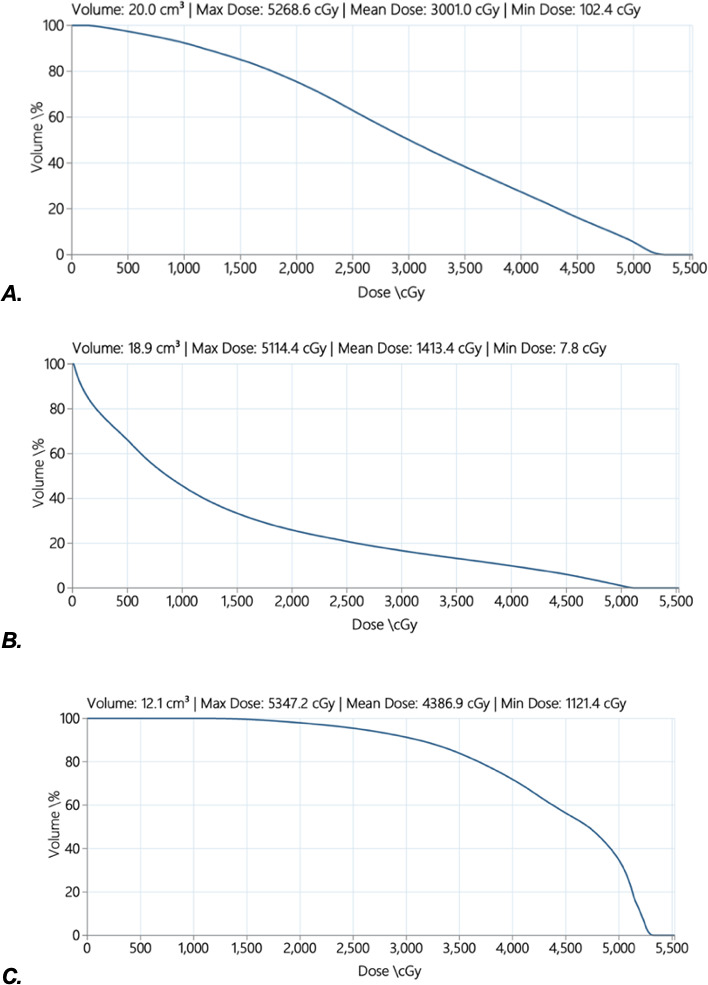
Dose-volume histogram (DVH) of the maxilla and the mandible. Doses given in Gy(RBE = 1.1). (A) DVH of the entire maxilla, D_mean_ 30 Gy. (B) DVH mandibular body, D_mean_ 14.1 Gy. (C) DVH mandibular rami, D_mean_ 43.9 Gy.

In this case, it was estimated that 75.4% of the maxilla received 20 Gy(RBE = 1.1) and 50% received 30 Gy(RBE = 1.1), with a D_max_ of 52.7 Gy(RBE = 1.1) (**Figure 5A**). The maxillary lateral sextants (left: mean 37.5 Gy[RBE = 1.1], right: mean 34.4 Gy[RBE = 1.1]) received a higher dose than the central maxillary sextant (mean 19.6 Gy[RBE = 1.1]). Despite the central maxillary sextant receiving a mean dose of < 20 Gy(RBE = 1.1), all teeth display DAs.

**Figure 5B** and **5C** show the DVH for the mandibular body and rami. DAs occurred in the mandible despite the lower mean dose (14.1 Gy(RBE = 1.1) compared to the maxilla and a lower dose than the proposed minimal tooth threshold of 20 Gy. The central mandibular sextant received a D_mean_ of 3 Gy(RBE = 1.1), which may explain the absence of dental toxicities in this region. However, despite the right lateral sextant receiving only a D_mean_ of 9.6 Gy(RBE = 1.1), there are disturbances of the LR5 and LR7.

## Discussion

In this patient, generalized DAs in the maxilla and mandible were noted 3.5-years posttreatment completion. This case supports statements that DAs are more common in children treated before the ages of 4 to 5 years [1, 18, 19]. The minimal observation period required to allow identification of DAs is unknown, yet in this case, the observation period exceeds the 2.5 years previously proposed [20]. It is difficult to assign the identified toxicities in this case to the grades outlined in the CTCAE, potentially highlighting the need to develop a more specific tool to classify DAs.

It could be suggested from the multiple disturbances identified in this case that the minimum dose threshold of 20 Gy (D_mean_) proposed in the Pediatric Normal Tissue Effects in the Clinic review [1] does not apply to all cases. However, most cohorts included in the Pediatric Normal Tissue Effects in the Clinic review were treated with conventional photon radiation therapy, arguably limiting its applicability to PBT. Therefore, a more detailed analysis of PBT radiation dose limits to dental structures is warranted.

The authors recognize the potential confounding factor of concomitant chemotherapy and therefore cannot conclude that the DAs are solely a result of PBT. The lack of discussion about the influence of chemotherapy is frequent in the PBT literature [19, 21–25]. In addition, it is challenging to compare different treatment schedules without detailed reporting of antineoplastic agents, doses, and duration. Furthermore, unless a clinical history and pretreatment dental radiographs are available, it is impossible to exclude genetic and environmental (eg, nutritional) causes of DAs, considering their multifactorial etiology [26].

Although delineation of organs at risk is part of routine practice, the maxilla is not contoured for all diagnoses. Although difficult in the primary dentition, an alternative approach would be to delineate individual teeth [27]. In this case, the findings suggest the maxilla should be routinely contoured and considered a low-priority avoidance structure along with the mandible in radiation therapy planning. This may allow for easier extraction of dosimetric data, thus helping to establish a dose-effect relationship and allowing improved counseling of patients on potential dentofacial sequelae.

Without detailed reporting of dental toxicities in pediatric cancer survivors, it is impossible to determine the impact of the radiation dose and age on the risk of DAs or ensure referral of children to the appropriate dental services [1, 9]. Long-term dental follow-up with the appropriate consideration of radiographs in those treated for pediatric head and neck malignancies is essential.

## Conclusion

Significant dental DAs occurred in this pediatric patient treated with a combination of PBT and chemotherapy for RMS when 3.5 years old. Prescribed doses as low as 9.6 Gy(D_mean_) in the mandible correlated with DAs. There is a need for ongoing work in identifying accurate dose thresholds for dental-bearing areas and contouring these areas routinely as avoidance structures. This will only be made possible with prospective collection of long-term clinical dental toxicities, correlated with dosimetric data and controlled for impactful treatments such as chemotherapy.
